# Acute T-cell leukemia/lymphoma presenting with leukemia cutis mimicking prurigo nodularis

**DOI:** 10.1016/j.jdcr.2026.03.014

**Published:** 2026-03-13

**Authors:** Magdi Elghannam, Muhammad Osto, Jennifer Strong, Marcia Driscoll

**Affiliations:** Department of Dermatology, University of Maryland School of Medicine, Baltimore, Maryland

**Keywords:** acute T-cell leukemia/lymphoma, ATLL (adult T-cell lymphoma/leukemia), HTLV-1 (human T-cell lymphotropic virus type 1), leukemia cutis, prurigo nodularis, pruritus, skin of color

## Introduction

Adult T-cell lymphoma/leukemia (ATLL) is a CD4^+^ T-cell neoplasm most commonly caused by human T-cell lymphotropic virus type 1 (HTLV-1).^1^ It can manifest into 4 clinical subtypes: acute, smoldering, chronic, and lymphoma.^1^ Initial presentation differs depending on the subtype of ATLL, often presenting with B-symptoms, hepatosplenomegaly, hypercalcemia, and elevated lactate dehydrogenase (LDH).^1^ ATLL is frequently associated with leukemia cutis, a condition characterized by the infiltration of neoplastic leukocytes into the skin.^2^ An estimated 50% of patients with ATLL develop leukemia cutis, which can serve as the heralding symptom of the malignancy.^2^ Although pruritus is a recognized symptom in ATLL, it is often mild and underemphasized in clinical descriptions.^3^ Severe, refractory pruritus at presentation remains infrequently reported and is rarely the dominant clinical complaint. We describe a patient with ATLL-associated leukemia cutis presenting as intensely pruritic papulonodular lesions clinically mimicking prurigo nodularis. In this case, malignant cutaneous infiltration produced nodular, excoriated lesions that resembled prurigo nodularis, which contributed to initial diagnostic delay. This presentation highlights how leukemia cutis can assume prurigo-like morphology when associated with intense pruritus. This case contributes to the growing recognition of leukemia cutis presenting with prurigo-like morphology and highlights the need for heightened diagnostic suspicion in HTLV-1-endemic populations.

## Case report

A 59-year-old man presented 5 months after an eruption of a diffuse pruritic rash. Initially when the eruption began, an outside dermatologist diagnosed the condition as folliculitis and prescribed a selenium sulfide wash. When the rash failed to improve, the patient's primary care physician administered a 10-day course of oral prednisone, which provided partial relief from the pruritus. However, despite these interventions, the skin lesions persisted. Upon evaluation at our medical center, the patient also exhibited significant weight loss, arthralgia, abdominal pain, and leukocytosis. While not appreciable on physical exam positron emission tomography computed tomography scan imaging revealed multiple enlarged retroperitoneal, mesenteric, axillary, and inguinal lymph nodes, along with splenomegaly, no skeletal or visceral organ involvement was noted. Laboratory investigations revealed a calcium level of 9.9 mg/dL (Reference range: 8.6-10.2 mg/dL), LDH level of 352 U/L (Reference range: 120-246 U/L), and peripheral eosinophilia (16%) (Reference range: 0%-6%). Infectious workup showed negative Strongyloides serology, and cytomegalovirus testing was equivocal.

**Fig 1:**
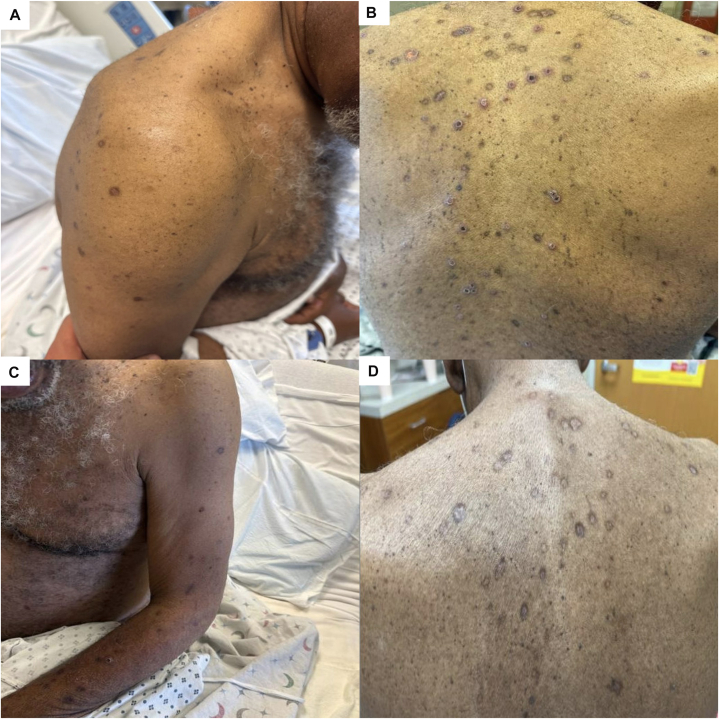
Diffuse papulonodular lesions with central erosion and crusting, distributed across the patient’s shoulders **(A)**, back **(B)**, upper extremities **(C)**. Healing annular lesion with peripheral hyperpigmentation, central hypopigmentation, and atrophic indentation (post-inflammatory atrophy), pictured 6 weeks after EPOCH therapy (etoposide, prednisone, vincristine, cyclophosphamide, doxorubicin) **(D)**.

**Fig 2:**
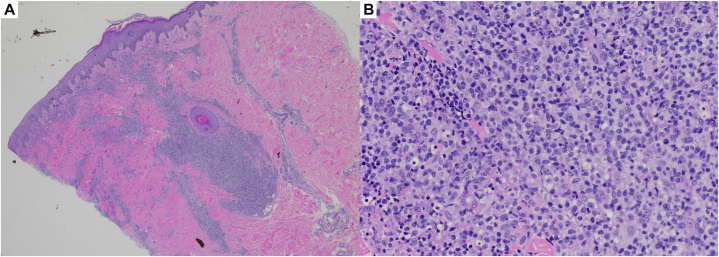
Punch biopsy of patient’s lesion. H&E stain **(A)** Low-power view (original magnification: ×100). **B,** High-power view highlighting cytologic atypia (original magnification: ×400).

**Fig 3:**
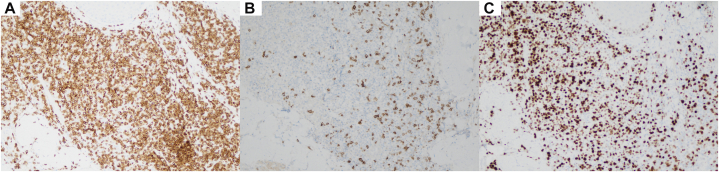
Immunohistochemical staining of the skin biopsy. **A,** CD3 highlighting a dense infiltrate of T-cells. **B,** Loss of CD7 expression in atypical lymphoid cells. **C,** High Ki-67 proliferation index.

**Fig 4:**
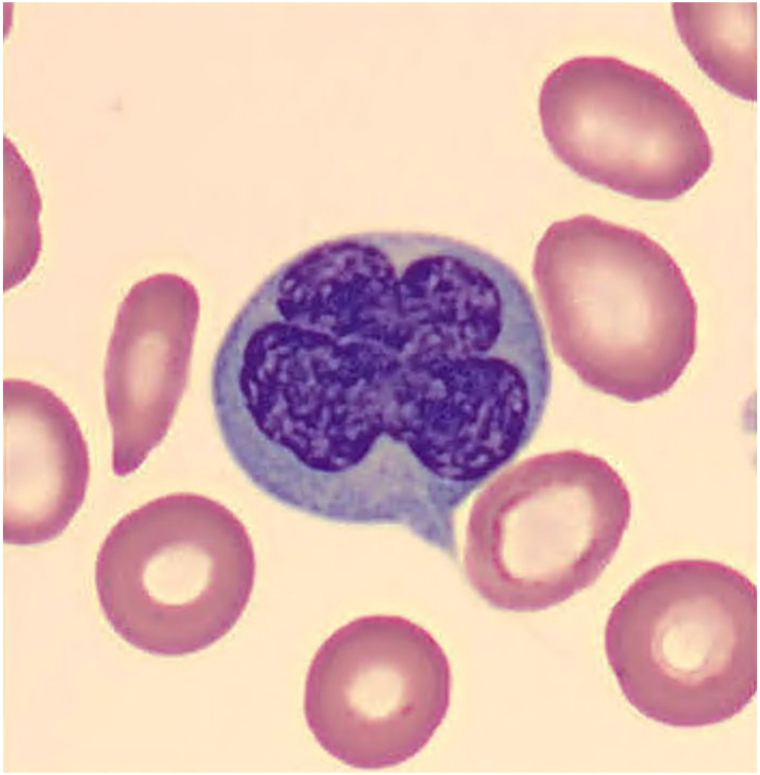
Atypical lymphocytes with multilobulated “floret-like” nuclei on peripheral smear.

On exam, diffuse, eroded, and centrally crusted papulonodular lesions were present on his trunk and bilateral upper and lower extremities (Fig 1, *A*-*C*). A punch biopsy from the patient’s back showed atypical cells with irregular nuclear borders, variable chromatin patterns, prominent nucleoli, and occasional atypical mitotic figures, alongside spongiosis and basal vacuolation in the epidermis (Fig 2, *A* and *B*). Immunohistochemistry demonstrated CD3 and CD25 positivity, CD7 loss, and a high Ki-67 index of 70 (Fig 3, *A*-*C*). Peripheral blood smear revealed leukocytosis and lymphocytosis with 56% atypical lymphocytes displaying characteristic “flower-like” nuclear contours (Fig 4). HTLV-1 serology was positive. Bone marrow biopsy showed hypercellularity, with 30% of the marrow space occupied by atypical lymphocytes. Based on these findings, a diagnosis of acute ATLL was established. The patient was initiated on EPOCH therapy (Etoposide, Prednisone, Oncovin, Cyclophosphamide, and Hydroxydaunorubicin). At 6-week follow-up, the patient’s skin rash demonstrated substantial improvement (Fig 1, *D*).

## Discussion

ATLL is diagnosed through a combination of clinical, laboratory, and histopathological findings. This patient exhibited several characteristic features, including HTLV-1 seropositivity, lymphocytes with “flower-like” nuclear contours, CD25 positivity, systemic B-symptoms.^1^ However, in some cases—such as the 1 presented—lymphoma-associated pruritus can precede other hallmark signs by months or even years and remains an underrecognized initial symptom.^4^ While up to 35% of patients with ATLL experience pruritus, it is often mild and nonspecific, leading to diagnostic delays.^3^

Early U.S. data from Bunn et al reported pruritus in just 9% of ATLL cases, all described as mild.^5^ This stark contrast with our case—characterized by severe pruritus with nodular leukemia cutis lesions that clinically mimicked prurigo nodularis—highlights possible regional, genetic, or viral subtype-based variability in presentation.

Given the high prevalence of HTLV-1 in regions such as southwestern Japan, sub-Saharan Africa, the Caribbean, South America, and parts of the Middle East,^6^ clinicians should maintain heightened awareness of atypical presentations—particularly in patients from endemic or immigrant populations. Severe, treatment-resistant pruritus should prompt evaluation for underlying malignancy, as timely diagnosis is critical in acute ATLL, which carries a median survival of less than 1 year.^7^

Leukemia cutis in ATLL can present in diverse morphologies, including papules, nodules, ulcers, and plaques, and may represent the disease’s initial clinical sign.^1^^,^^4^ Its presence is frequently associated with systemic involvement and portends a poor prognosis.^1^ Diagnosis typically involves a skin biopsy, and management centers on treating the underlying malignancy.^1^ Although the pathogenesis of pruritus in T-cell neoplasms remains unclear, proposed mechanisms include Th2 cytokine dysregulation and eosinophilic infiltration, which may also explain the patient’s peripheral eosinophilia despite a negative infectious workup.^8^

While standard chemotherapy regimens like EPOCH remain a mainstay, novel targeted therapies are emerging and showing promise. Mogamulizumab, a humanized monoclonal antibody targeting CC chemokine receptor 4 (CCR4)—frequently expressed on ATLL cells—has demonstrated improved progression-freesurvival in clinical trials and is approved for ATLL treatment in several countries, including Japan, the US, and Canada.^9^^,^^10^ Lenalidomide, an immunomodulatory agent, has shown antitumor activity through T-cell costimulation and inhibition of pro-inflammatory cytokines.^9^ Additionally, brentuximab vedotin, an anti-CD30 antibody-drug conjugate, offers potential benefit in CD30-positive ATLL subtypes and is currently under investigation.^9^ Together, these agents represent a promising shift toward more personalized, immune-targeted approaches in ATLL management.

This case underscores a rare but important clinical entity: ATLL-associated leukemia cutis presenting as intensely pruritic nodular lesions that mimic prurigo nodularis. While based on a single patient with limited follow-up, it highlights how recognizing such unusual cutaneous manifestations may not only prevent delays in life-saving therapy but also refine our understanding of ATLL’s dermatologic spectrum across diverse populations.

## Conflicts of interest

None disclosed.
